# Unraveling inner experiences during resting state

**DOI:** 10.3389/fnhum.2013.00409

**Published:** 2013-07-29

**Authors:** Juergen Fell

**Affiliations:** Cortical Oscillations Lab, Department of Epileptology, University of BonnBonn, Germany

The investigation of resting state activity has become an extremely busy topic in neurocognitive research. A recent pubmed search revealed more than 2000 entries for the terms “resting state” AND “fMRI.” Several putative clinical applications have been suggested, such as the detection of early signs of Alzheimer's disease (e.g., Lustig et al., 2003) or risk of developing psychosis (e.g., Jukuri et al., 2013). However, the usefulness of resting state data is hampered by the fact, that up to now only very few investigations have tried to elucidate the mental processes occurring during these experiments. Without knowledge of these processes the purely physiologically based findings of altered activations and functional connectivities during resting state lack a meaningful neurocognitive interpretation.

Today, more and more knowledge accumulates that our minds frequently wander during daytime activities and create thoughts which are not directly associated with the situation at hand (e.g., Schooler et al., 2011). These thoughts are, for instance, related to autobiographical memories, future planning, social interactions, and problem-solving. By exploiting the technique of so-called experience sampling, it has been demonstrated that such mind wandering occupies up to 50% of our daytime experiences (Killingsworth and Gilbert, 2010). During times when no task performance is required, such mind wandering is even more prevalent. Thus, resting state activity may veridically be described as reflecting not an idling state, but rather a “free thinking” state (Everding, 2003). Accordingly, the patterns of resting state activity, such as activations of so-called default-mode regions, are probably strongly related to the contents of these thoughts (e.g., Mason et al., 2007). Individual differences in the frequency and contents of mind wandering may thus correspond to discrepant patterns of resting state activity and even may lead to systematic biases, for instance, caused by age (e.g., Maillet and Rajah, 2013) or population (e.g., Song and Wang, 2012) related differences.

Recent studies have moreover shed light on the more general question, which kinds of inner experiences are prevalent in humans, i.e., experiences that occur in addition to the permanent sensory monitoring of the external environment (e.g., Heavey and Hurlburt, 2007; Hurlburt, 2011). Based on the technique of so-called descriptive experience sampling and investigations of several hundreds of subjects, a catalog of these inner experiences has been suggested (Hurlburt and Heavey, 2006). According to these studies (e.g., Heavey and Hurlburt, 2007) the most common forms of inner experiences are “inner seeing” (i.e., experiencing inner images), “feelings,” “inner speech” (constituting a certain kind of expression of thought), “unsymbolized thinking” (i.e., thinking which is not conveyed in words, images or other symbols) and “sensory awareness” (i.e., paying attention to a particular sensory aspect of the environment). With the exception of the latter category (sensory awareness) these inner experiences again are closely related to mind wandering.

A particularly interesting outcome of these studies is, that the distribution of the categories of inner experiences has an extremely high interindividual variability across individuals (e.g., Heavey and Hurlburt, 2007)—even though we humans experience the external environment in a rather similar fashion. There are, for instance, subjects, who mainly experience inner seeing, but very rarely inner speech, whereas for other individuals the opposite is true. Accordingly, the individual patterns of resting state activity may strongly depend on the categories of inner experiences, which are prevalent in a certain subject. Alterations of a subject's resting state patterns as compared to an average collective sample may thus be largely determined by her/his individual characteristics of inner experiences. This interindividual variability, of course, seriously impairs the clinical usefulness of such data, for instance, in the detection of early Alzheimer's disease.

Following on from these considerations, it is of paramount importance to access the categories and contents of inner experiences occurring during resting state experiments. A fundamental methodological difficulty complicating this endeavor is that we are typically unaware of our mind wandering and inner experiences. In other words, we commonly lack metaawareness of these experiences (Schooler, 2002). This is the case even, for instance, when subjects know their mind wandering during a sustained attention task will be sampled (Christoff et al., 2009). Interestingly, mind wandering related activations are stronger when subjects are unaware as compared to when they are aware of these experiences (Christoff et al., 2009). Accordingly, the frequency distribution of absent versus present meta-awareness represents another individual factor determining resting state patterns.

Related to the common lack of metaawareness for inner experiences is the prevailing inability to remember these experiences (research addressing this interrelation is yet also lacking), which makes them difficult to reconstruct by later inquisition. Actually, it has been suggested to inquire about inner experiences during resting state fMRI using a *post-hoc* questionnaire (Delamillieure et al., 2009). This questionnaire comprised of categories such as “visual imagery,” “inner language,” “somatosensory awareness,” “inner musical experiences,” and “mental processing of numbers,” as well as several items addressing the contents of these experiences. Based on this questionnaire, for instance, functional connectivity during resting state has been analyzed depending on the subject's self-reported time spent thinking in mental images and inner language (Doucet et al., 2012). Although this pioneering work is to be complimented, it remains doubtful whether subjects are indeed able to reliably identify their inner experiences after a temporal delay of several minutes. Other data suggest that the outcome of such *post-hoc* inquiries in subjects, which are naïve to the monitoring of inner experiences, can generally not be trusted and, for instance, strongly depends on the subject's preconceptions and beliefs concerning their inner experiences (e.g., Schooler, 2002; Hurlburt, 2011).

A more reliable approach to access inner experiences during resting state experiments may be to use reports, which are elicited *ad-hoc* via probe stimuli. Compared to self-elicited reports, i.e., when subjects give an account whenever they notice the occurrence of an inner experience, probe-elicited reports offer two major advantages. First, because of the elusiveness of inner experiences, it is generally less difficult to describe these experiences when prompted by a probe than to provide self-elicited reports (Hurlburt, 2011; Schooler et al., 2011). Second, the time intervals occupied by meta-cognitive monitoring are more clearly defined in the probe elicited condition (basically the times between the probes and the responses) compared to the self-elicited condition. Since these time intervals in a strict sense may not be labeled as task-free “resting state,” they may later be excluded from the evaluation of resting state activity.

In its simplest form, for instance, with this approach the most common categories (e.g., inner seeing, feeling, inner speech, other) and contents (e.g., past, future, about the experiment, other) of inner experiences would be sampled. These inquiries could be implemented, for example, by a pair of visually prompted subsequent questions requiring button presses with a beep tone accompanying the first question (e.g., “What kind of inner experience did you have just before the beep”; “What was the content of this experience related to?”). Subjects should be allowed to give more than one response to each question (e.g., to report the experiences of inner seeing plus inner speech). Repeated pairs of questions should be separated by randomized time intervals (e.g., 50–70 s). Of course, the suggested method can reveal only a coarse-grained picture of inner experiences occurring during resting state and the few probe-elicited reports have to be extrapolated for the complete experiment.

For several disease populations, group differences in resting state measures compared to healthy controls have been detected, which may help to understand the pathophysiological bases of these diseases (e.g., Lustig et al., 2003; Lee et al., 2012; Jukuri et al., 2013). It has been pointed out, however, that these findings are of little use in individual diagnosis, endophenotype development, prognosis and treatment monitoring (e.g., Anderson et al., 2011). From a practical standpoint, a robust classification of single-subject resting state measures with regard to control and disease collectives would be desirable, which, in the ideal case, could back up clinical decisions. To accomplish this goal, it is necessary to investigate the reliability of such single-subject classifications and to reduce any major variance introduced by thus far uncontrolled factors. Since it has been demonstrated that resting state measures considerably depend on individual inner experiences during these experiments, for instance, on the estimated amount of time spent thinking in mental images or inner language (Doucet et al., 2012), taking experience sampling based variables into account may have the potential to substantially enhance the validity of such classifications.

To conclude, similar to the exploitation of first-person data to elucidate the variability of neural responses during cognitive experiments (Lutz et al., 2002), accessing the categories and contents of inner experiences via the suggested basic procedure may enable researchers to explain a large amount of interindividual variance in resting state data. Hence, this approach may have the potential to greatly improve the usefulness of resting state experiments for clinical purposes.
